# Urinary extracellular vesicles as a source of protein‐based biomarkers in feline chronic kidney disease and hypertension

**DOI:** 10.1111/jsap.13536

**Published:** 2022-07-07

**Authors:** J. S. Lawson, H. M. Syme, P. R. Antrobus, J. M. Karttunen, S. E. Stewart, F. E. Karet Frankl, T. L. Williams

**Affiliations:** ^1^ Clinical Sciences and Services, The Royal Veterinary College Hatfield AL9 7TA UK; ^2^ Cambridge Institute for Medical Research University of Cambridge, Keith Peters Building Cambridge CB2 0XY UK; ^3^ Department of Veterinary Medicine, The Queen's Veterinary School Hospital University of Cambridge Cambridge CB3 0ES UK; ^4^ Department of Biochemistry and Genetics, La Trobe Institute for Molecular Science La Trobe University Bundoora Victoria 3086 Australia; ^5^ Department of Medical Genetics and Division of Renal Medicine University of Cambridge and Cambridge University Hospitals Foundation Trust Cambridge UK

## Abstract

**Objectives:**

To validate a methodology for isolating feline urinary extracellular vesicles and characterise the urinary extracellular vesicle population and proteome in cats with normal renal function and cats with normotensive or hypertensive chronic kidney disease.

**Methods:**

Feline urinary extracellular vesicles were isolated using three different methods (precipitation alone, precipitation followed by size exclusion chromatography and ultrafiltration followed by size exclusion chromatography, which were compared via transmission electron microscopy and nanoparticle tracking analysis. Cats with normal renal function (n=9), normotensive chronic kidney disease (n=10) and hypertensive chronic kidney disease (n=9) were identified and urinary extracellular vesicles isolated from patient urine samples via ultrafiltration followed by size exclusion chromatography. Extracellular vesicle size and concentration were determined using nanoparticle tracking analysis, and subsequently underwent proteomic analysis using liquid chromatography with tandem mass spectrometry to identify differences in protein expression between categories.

**Results:**

Urinary extracellular vesicle preparations contained particles of the expected size and morphology, and those obtained by ultrafiltration + size exclusion chromatography had a significantly higher purity (highest particle: protein ratio). The urinary extracellular vesicle proteomes contained extracellular vesicle markers and proteins originating from all nephron segments. Urinary extracellular vesicle concentration and size were unaffected by renal disease or hypertension. There were no differentially expressed proteins detected when comparing urinary extracellular vesicles derived from cats in the healthy category with the combined chronic kidney disease category, but five differentially expressed proteins were identified between the normotensive chronic kidney disease and hypertensive chronic kidney disease categories.

**Clinical Significance:**

Feline urinary extracellular vesicles can be successfully isolated from stored urine samples. Differentially expressed urinary extracellular vesicle proteins were discovered in cats with hypertensive chronic kidney disease, and warrant further investigation into their utility as biomarkers or therapeutic targets.

## INTRODUCTION

Chronic kidney disease (CKD) is common in geriatric cats, with a reported prevalence of up to 50% (Marino *et al*. 2014). In the majority of cases, where tubulointerstitial fibrosis is the predominant pathology, the underlying aetiology is not understood (Chakrabarti *et al*. 2013). CKD is associated with hypertension in approximately 20% of cats, which has the potential to significantly contribute to the morbidity and mortality associated with this condition (Syme *et al*. 2002). Although several mechanisms for this association have been suggested, the precise relationship between CKD and hypertension in cats has not been fully elucidated (Lawson & Jepson 2021). There is currently no effective treatment aside from dietary alteration that significantly slows the progression of renal disease in cats, and considerable variation in disease progression exists. Identifying factors involved in the pathogenesis of CKD and hypertension would be of significant value to the feline population in order to identify biomarkers of disease progression and potential therapeutic targets.

Extracellular vesicles (EVs) are a heterogenous group of membranous structures actively released from cells into their surroundings, that contain proteins, mRNAs and microRNAs (Svenningsen *et al*. 2020). Urinary EVs are a rich source of potential biomarkers, since they originate primarily from the kidney and urinary tract epithelium, and have previously been demonstrated to contain apical proteins from all nephron segments (Svenningsen *et al*. 2020). EVs are also relatively resistant to proteinases and RNAses, and proteins present within (or associated with) EVs are protected from tubular reabsorption. In humans, the protein signature of EVs isolated from the urine of patients with tubulopathies (Corbetta *et al*. 2015, Williams *et al*. 2020), acute kidney injury (du Cheyron *et al*. 2003) and glomerulonephropathies (Burger *et al*. 2014) can differentiate these patients from healthy controls.

Previous studies have demonstrated that isolation of EVs from feline urine is feasible, and that significant alterations in the expression of microRNAs associated with EVs are detectable in cats with CKD (Ichii *et al*. 2018, Li *et al*. 2021). However, there are currently no studies characterising the feline urinary extracellular vesicle (uEV) proteome. This proteome, therefore, represents an as yet unexplored reservoir which could provide novel insights into the pathogenesis of feline CKD and hypertension, potentially leading to the identification of biomarkers and novel drug targets. Furthermore, cataloguing the EV proteome of individual cats could provide diagnostic information analogous to a “liquid renal biopsy”.

The aim of this study was to validate a methodology for isolating feline uEVs and to characterise the uEV proteome in cats with normal renal function in comparison to cats with normotensive or hypertensive CKD. It was hypothesized that, as in humans, feline uEVs will contain proteins from all sections of the nephron, and as such are a reservoir for biomarker discovery. In addition, it was hypothesized that the uEV proteome will be altered in cats with CKD with and without hypertension, and that these alterations will provide novel insights into the pathogenesis of these conditions.

## METHODS

### Pilot experiment to determine an optimal methodology for the isolation of feline uEVs

#### Study design and inclusion criteria

In an initial experiment, feline uEVs were isolated from pooled aliquots of frozen urine (4 mL) via three different methods: precipitation, precipitation followed by size exclusion chromatography and ultrafiltration followed by size exclusion chromatography before EV isolation urine samples were thawed on ice, filtered through a 0.22‐μm filter and 80 μL of protease inhibitor [Sigma‐Aldrich; single tablet diluted in 1 mL phosphate‐buffered saline (PBS)] was added to each 4 mL aliquot. Precipitation was performed using a commercially available volume‐excluding polymer kit as per the manufacturer's protocol (miRCURY Exosome Cell/Urine/CSF Kit; Qiagen). The miRCURY exosome precipitation buffer (1.6 mL; Qiagen) was added to each sample before vortexing for 5 seconds. The resultant sample/precipitation buffer mixture was incubated overnight at 4°C, then centrifuged at 3200*g* for 30 minutes and the supernatant discarded. The pellet was centrifuged at 3200*g* for a further 1 minute, and any additional supernatant discarded, before being resuspended in either 600‐μL PBS (precipitation) or 150‐μL PBS (precipitation + size exclusion chromatography). Ultrafiltration was accomplished by centrifuging 4 mL urine aliquots in a 100 kDa concentrator column (Millipore) at 4000*g* for 5 minutes. This resulted in a final concentrated urine volume of approximately 100 μL. Size exclusion chromatography was performed using commercially available columns (qEV single 70 nm; IZON) as per the manufacturer's protocol. Briefly, buffer was removed from the columns which were then washed with PBS before instilling the resuspended precipitate or ultrafiltered urine, which was then followed by elution with PBS. The first 1000‐μL fraction was discarded, and the following 600 μL collected as the EV rich fraction, as previously optimised (Karttunen *et al*. 2021). Final uEV preparations were stored at −80°C until further analysis.

#### Outcomes

EV characterisation was performed according to the MISEV 2018 guidelines (Thery *et al*. 2018), although due to lack of species specific antibodies the presence of EV specific proteins were confirmed with proteomic analysis rather than western blotting. Transmission electron microscopy was undertaken to confirm the particles identified were consistent with EVs. Glow‐discharged electron microscopy grids were placed on a 5‐μL droplet of the sample for 2 minutes. The grids were washed twice by transferring them to a fresh droplet of distilled water for 2 minutes per wash. Excess fluid was removed with filter paper and the grids were transferred to one drop of uranyl acetate (1.5%) for 1 minute. Excess fluid was again removed and the grids were stored for imaging. Grids were imaged on a Tecnai G2 transmission electron microscope.

Nanoparticle tracking analysis was performed to confirm the size and concentration of EVs eluted. The uEV preparations were diluted in PBS to concentrations in the range of 10^8^ to 10^9^ particles/mL and analysed by nanoparticle tracking analysis on a Nanosight NS300 (Malvern) to confirm the size and concentration of eluted particles using NTA 3.2 software. Measurements were carried out using flow mode and a minimum of three 60‐second videos were recorded per independent sample. Camera gain, focus and detection threshold were kept constant for each experiment.

The protein content of uEV preparations was assessed using a commercially available bicinchoninic acid (BCA) protein assay kit (Pierce™ BCA Protein Assay; Thermo Fisher Scientific). An eight step standard curve was constructed from serial dilutions of bovine serum albumin (25 to 2000 μg/mL). Standards and samples (5 μL/well) were assayed in a clear 96‐well plate (Thermo Fisher Scientific) in duplicate, and incubated with 200 μL reagent for 30 minutes at 37°C. After incubation, absorbance was read at 562 nm using a microplate reader (M200 Pro Plate Reader; Tecan) and protein concentration of each experimental sample was determined from the standard curve. A particle: protein ratio (particles per μg protein) was calculated using these data and the nanoparticle tracking analysis results as a measure of purity (Webber & Clayton 2013).

### Characterisation of the uEV population in cats with normal renal function, normotensive CKD and hypertensive CKD

#### Study design and inclusion criteria

The electronic records of cats attending a geriatric cat clinic at two first‐opinion practices were searched for cats above 9 years old with and without CKD (normal kidney function). CKD was diagnosed based upon a persistently elevated plasma creatinine concentration of more than 177 μmol/L with concurrent urine specific gravity (USG) of less than 1.035. Cats were identified as having normal kidney function if plasma creatinine was less than 177 μmol/L and USG more than 1.035. Systolic blood pressure (SBP) was measured by the Doppler method as per ACVIM consensus guidelines and mean SBP calculated from five readings (Acierno *et al*. 2018). Systemic hypertension was defined as a SBP of more than 160 mmHg on two separate occasions, or a SBP of more than 160 mmHg on a single occasion alongside documented ocular target organ damage. Cats with hyperthyroidism (plasma total T4 > 40 nmol/L), urinary tract infection (evidence of bacteriuria/pyuria or positive urine culture), or evidence of other systemic disease on physical examination/serum biochemistry were excluded.

Blood and urine samples were obtained by jugular venepuncture and cystocentesis respectively. Urine sediment examination was performed in‐house. Plasma biochemistry and urine creatinine concentration were measured at an external laboratory (IDEXX Laboratories). Residual samples of urine and plasma were retained for research use with owner informed consent. All urine samples were centrifuged and stored at −80°C. The project protocol, owner information sheet and consent forms were approved by ANONYMISED.

Eligible cats were divided based on renal function and SBP into three categories: normal renal function, normotensive CKD, and hypertensive CKD. Frozen patient urine samples (4 mL) were thawed on ice before the addition of protease inhibitor (80 μL), and uEVs subsequently isolated via the previously described ultrafiltration + exclusion chromatography method.

#### Outcomes

EV concentration (per mL and normalised to urine creatinine) and mean EV diameter were determined using nanoparticle tracking analysis as previously described.

For proteomic analysis, samples were incubated using 1 mL ice‐cold acetone overnight at −20°C. Proteins thus precipitated were pelleted by centrifugation at 13,500 rpm, and pellets resuspended in 50 μL NuPAGE LDS sample buffer (Thermo Fisher Scientific). Samples were boiled and 25 μL loaded onto precast NuPAGE 4% to 12% Bis‐Tris polyacrylamide gel (Thermo Fisher Scientific) and electrophoresed approximately 1.5 cm at 75 V. The gels were washed and stained (SimplyBlue SafeStain; Thermo Fisher Scientific) before each lane was excised and cut into two equal sized pieces. These were fully destained and the proteins reduced, alkylated and trypsin‐digested in‐gel overnight. The resulting tryptic peptides were extracted, desiccated and resuspended in mass spectrometry solvent (0.1% TFA, 3% MeCN) for liquid chromatography with tandem mass spectrometry (LC‐MS/MS) analysis.

LC‐MS/MS was performed on a Q Exactive Plus interfaced with an EASY‐spray source coupled to an RSLC3000 nanoUPLC (all Thermo Fisher Scientific). Tryptic peptides were fractionated using a 50 cm C18 PepMap column (Thermo Fisher Scientific) maintained at 40°C with solvent A (0.1% formic acid) and solvent B (80% MeCN, 0.1% formic acid), using a gradient rising from 3% to 40% solvent B between 7 and 52 minutes followed by a wash at 95% B and 25 minutes column re‐equilibriation at 3% solvent A. A spray voltage of 1.3 kV was used with MS spectra acquired from 400 to 1500 *m*/*z* at 70,000 resolution. MS/MS spectra were acquired in a top 10 fashion at 17,500 resolution with a target ACG of 1×10^5^ and a maximum injection time of 250 milliseconds. Data were processed using MaxQuant v.2.0.1.0 with *Felis catus* and *Homo sapiens* databases with label‐free quantitation and iBAQ enabled.

### Statistical analysis

Data were assessed for normality using the Shapiro–Wilk test, and parametric or non‐parametric statistics used as appropriate. Particles per ml and particle: protein ratio were compared between methods utilised in the pilot experiment using one‐way analysis of variance (ANOVA) and post hoc Tukey's multiple comparison test (results reported as mean ±standard deviation). Age, creatinine, USG and SBP of cats included in the experiment to characterise the uEV proteome were compared between categories using the one‐way ANOVA with post hoc Tukey test, and sex distribution using the chi‐squared test. EV concentration (per mL and normalised to urine creatinine) and mean EV diameter were compared between categories using the Kruskal‐Wallis test [results reported as median (25th, 75th percentiles)].To gain functional insights into the uEV proteome, Gene Ontology analysis was performed using the PANTHER classification system (v.14.0). Differences in protein expression between categories (normal renal function *versus* all CKD cats and normotensive CKD *versus* hypertensive CKD cats) were calculated using the unpaired Student's *t*‐test with Benjamini, Krieger and Yekutieli false discovery rate [reported as FDR adjusted P values reported (Q)]. To represent these *t*‐test data graphically, volcano plots illustrating log10 (FDR) *versus* log2 difference were constructed.

## RESULTS

### Pilot experiment to determine optimal methodology for the isolation of feline uEVs

**FIG 1:**
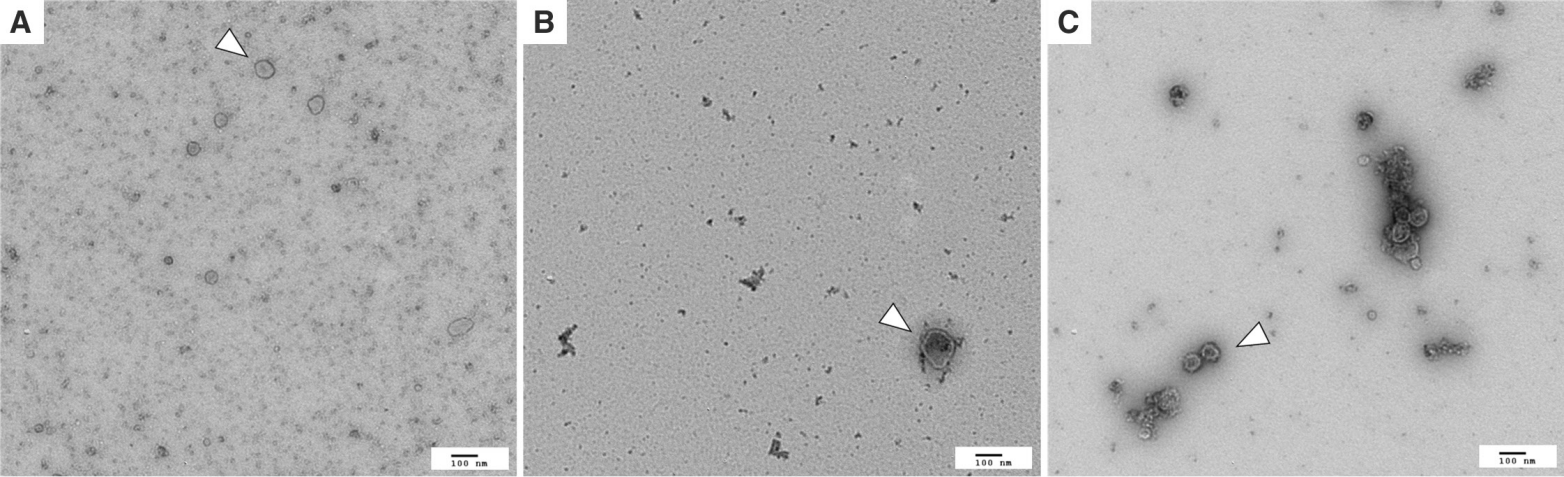
Transmission electron microscopy images of uEVs (white arrow) isolated via (A) precipitation (B) precipitation + size exclusion chromatography (SEC) and (C) ultrafiltration + SEC at ×11,500 magnification. Particles with a “cup‐shaped” morphology and within the expected size range were present in all preparations. The preparations obtained by precipitation alone contained a large amount of background particulate matter, considered to represent protein aggregates. The preparations obtained via ultrafiltration + SEC and precipitation + SEC contained less contamination, however only sparse vesicles were present in the precipitation + SEC samples

**FIG 2:**
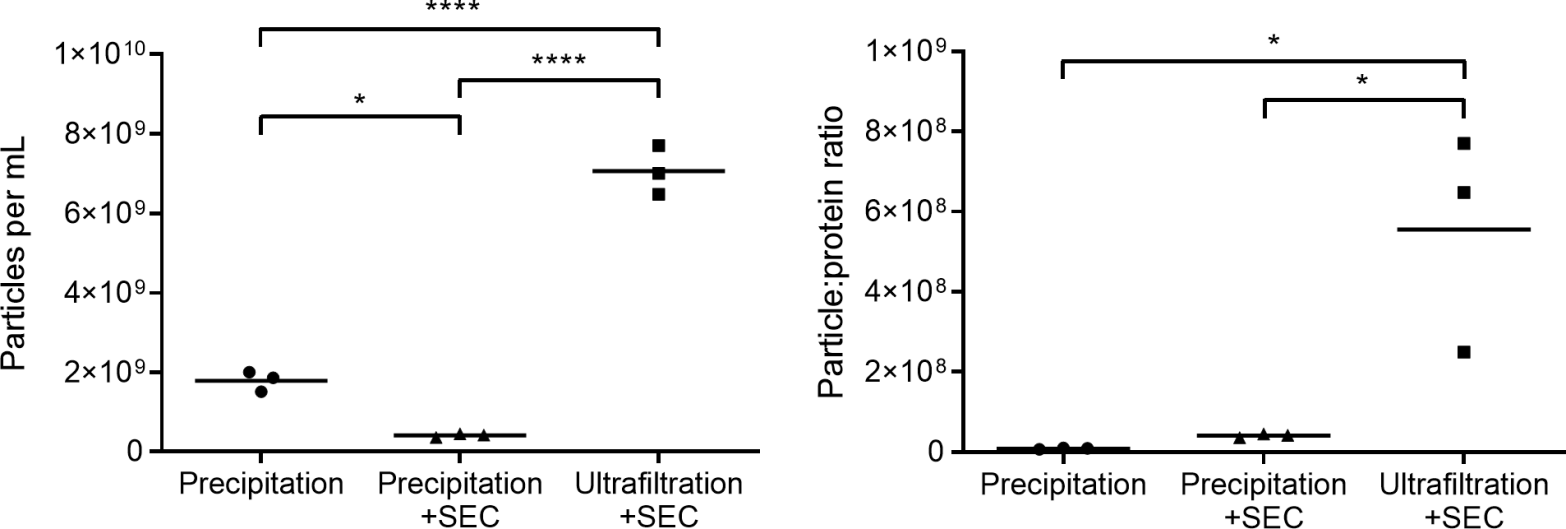
Scatterplot illustrating the concentration of particles per mL (left) and particle:protein ratio (purity) of feline uEV preparations (right) obtained via the three isolation methods precipitation, precipitation + size exclusion chromatography (SEC), ultrafiltration + SEC from a single sample of pooled feline urine. There were a significantly higher mean number of particles detected per mL by NTA in the uEV preparations obtained via ultrafiltration + SEC (7.1×10^9^ ±6.1×10^8^) *versus* uEV preparations obtained by precipitation alone (1.8×10^9^ ±2.5×10^8^; P<0.0001) or precipitation + SEC (4.1×10^8^ ±4.8×10^7^; P<0.0001). The difference in particle number between the preparations obtained by precipitation alone and precipitation + SEC was also significant (P=0.01). uEV preparations obtained via ultrafiltration + SEC also had a significantly higher mean particle to protein ratio (5.6×10^8^ ±2.7×10^8^) than preparations obtained via precipitation alone (9.0×10^6^ ±1.9×10^6^; P=0.013) or precipitation + SEC (4.1×10^7^ ±4.8×10^6^; P=0.017). Lines represent the median. *P<0.05, ****P<0.0001

The uEV preparations obtained via all three methods contained particles of the expected size and characteristic “cup‐shaped” morphology on transmission electron microscopy (Fig 1). Those preparations obtained by precipitation alone contained a large amount of background particulate matter, considered to represent protein aggregates. The preparations obtained via ultrafiltration + size exclusion chromatography and precipitation + size exclusion chromatography contained visibly less contamination, however only sparse vesicles were present in the precipitation + size exclusion chromatography samples. A significantly higher mean number of particles was detected per mL by nanoparticle tracking analysis in the uEV preparations obtained via ultrafiltration + size exclusion chromatography (7.1×10^9^ ±6.1×10^8^) *versus* uEV preparations obtained by precipitation alone (1.8×10^9^ ±2.5×10^8^, P<0.0001) or precipitation + size exclusion chromatography (4.1×10^8^ ±4.8×10^7^, P<0.0001; Fig 2). The difference in particle number between the preparations obtained by precipitation alone and precipitation + size exclusion chromatography was also significant (P=0.01). uEV preparations obtained via ultrafiltration + size exclusion chromatography had a significantly higher particle to protein ratio (5.6×10^8^ ±2.7×10^8^) than preparations obtained via precipitation alone (9.0×10^6^ ±1.9×10^6^, P=0.013) or precipitation + size exclusion chromatography (4.1×10^7^ ±4.8×10^6^; P=0.017; Fig 2). Mean differences and 95% confidence intervals for these comparisons may be found in Data S1.

### Characterisation of the uEV population and proteome in cats with normal renal function, normotensive CKD and hypertensive CKD

**FIG 3:**
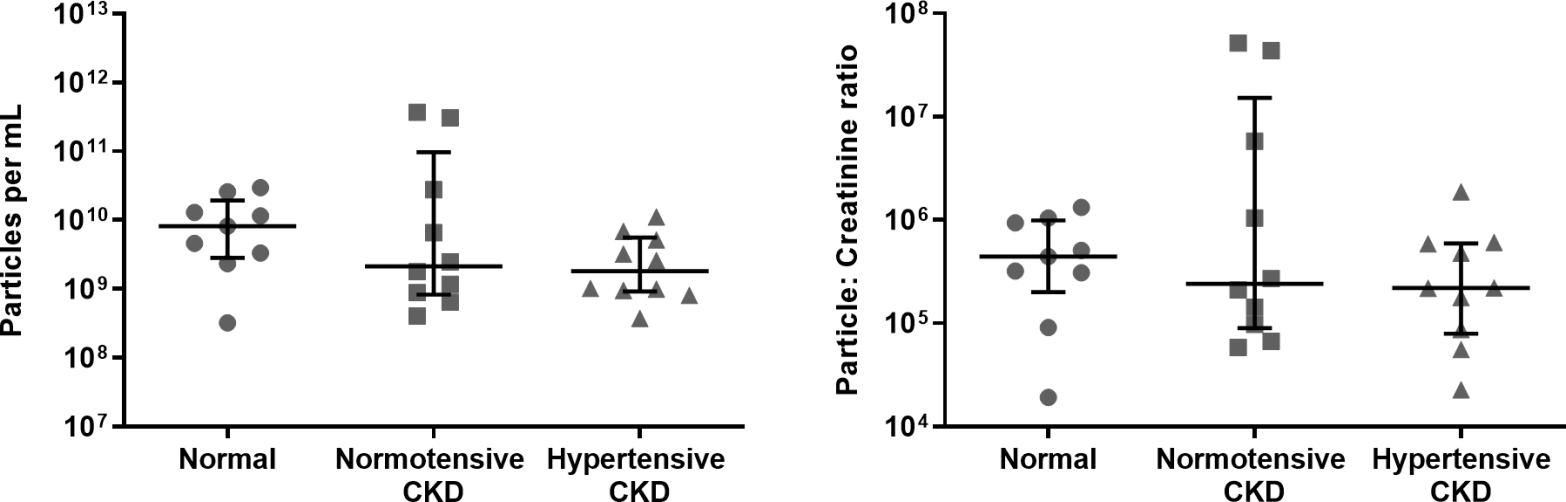
Scatterplot illustrating uEV particles per ml of urine and normalised to urine creatinine concentration, as measured by nanoparticle tracking analysis. There was no significant difference in uEV concentration per mL between cats with normal renal function [8.1×10^9^ (2.8×10^9^, 1.9×10^10^) particles/mL], normotensive CKD [2.1×10^9^ (8.3×10^8^, 9.8×10^10^) particles/mL] and hypertensive CKD [1.8×10^9^ (9.2×10^8^, 5.6×10^9^) particles/mL; P=0.26]. There remained no significant difference between category when EV concentration per mL was normalised to urine creatinine (P=0.68). Lines represent median, and bars the interquartile range

**FIG 4:**
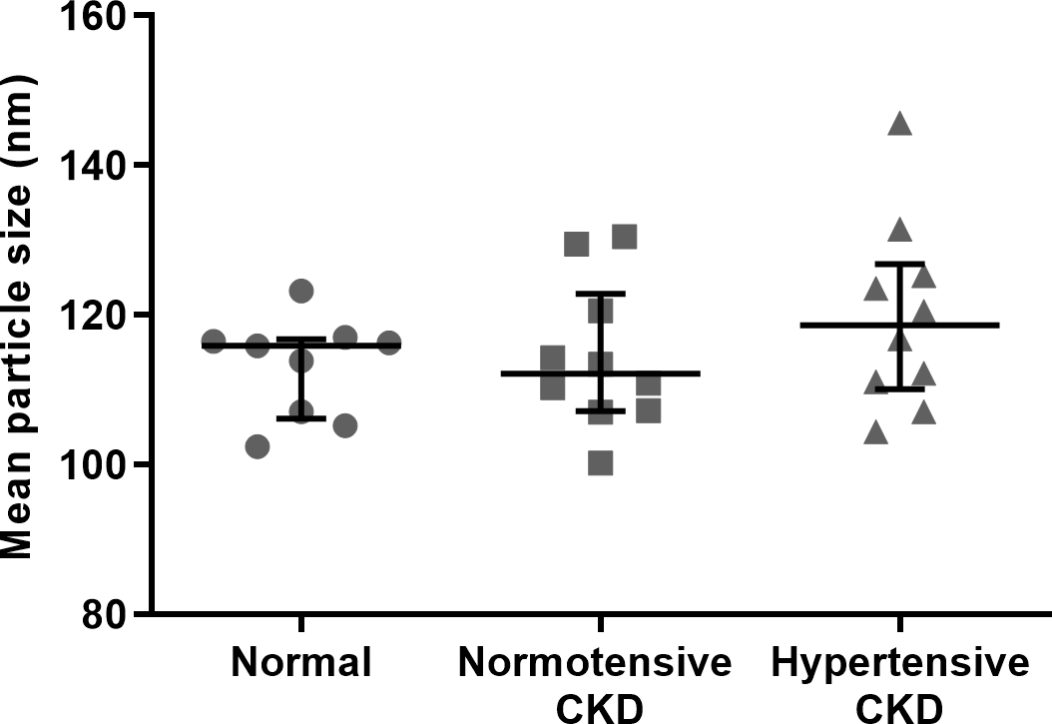
Scatterplot illustrating mean particle size (nm) of uEVs, as measured by nanoparticle tracking analysis. Mean particle size did not differ between category [normal renal function: 115.9 (106.2, 116.8) nm, normotensive CKD: 112.2 (107.2, 122.8) nm, hypertensive CKD 118.7 (110.1, 126.9) nm, P=0.42]. Lines represent median, and bars the interquartile range

Twenty‐eight cats were included in the study to characterise the feline uEV population: nine in the normal category, 10 with normotensive CKD and nine with hypertensive CKD. Summary data for these categories can be found in Table 1. There was no significant difference in uEV concentration (normalised per mL of urine) between cats with normal renal function [8.1×10^9^ (2.8×10^9^, 1.9×10^10^) particles/mL], normotensive CKD [2.1×10^9^ (8.3×10^8^, 9.8×10^10^) particles/mL] and hypertensive CKD [1.8×10^9^ (9.2×10^8^, 5.6×10^9^) particles/mL; P=0.26; Fig 3]. No significant difference emerged between categories when EV concentration per mL was instead normalised to urine creatinine concentration (P=0.68; Fig 3). Mean particle size did not differ between category [normal renal function: 115.9 (106.2, 116.8) nm, normotensive CKD: 112.2 (107.2, 122.8) nm, hypertensive CKD 118.7 (110.1, 126.9) nm; P=0.42; Fig. 4]. Mean rank differences for these comparisons may be found in Data S1.

**Table 1:** Summary data for cats included in the study to characterise the feline uEV population and proteome

	Normal	Normotensive CKD	Hypertensive CKD	P
Age (years)	12.0 (9.5 to 14.1)†	15.7 (9.5 to 20.5)†	15.0 (12.1 to 17.1)†	0.06
Creatinine (μmol/L)	103 (84 to 154)†	235 (178 to 615)‡	241 (187 to 286)‡	<0.0001
USG	1.048 (1.038 to 1.060)†	1.017 (1.013 to 1.030)‡	1.016 (1.014 to 1.027)‡	<0.0001
Blood pressure (mmHg)	127 (112 to 154)†	143 (124 to 156)†	203 (184 to 300)‡	<0.0001
Sex distribution (all neutered)	4 M, 5 F†	7 M, 3 F†	4 M, 5 F†	0.43

Values reported represent the median (range)

uEV urinary extracellular vesicle, USG urine specific gravity, M Male, F Female

^†^ Within a row, medians without a common superscript differ

^‡^ Medians without a common superscript differ (P<0.05)

**FIG 5:**
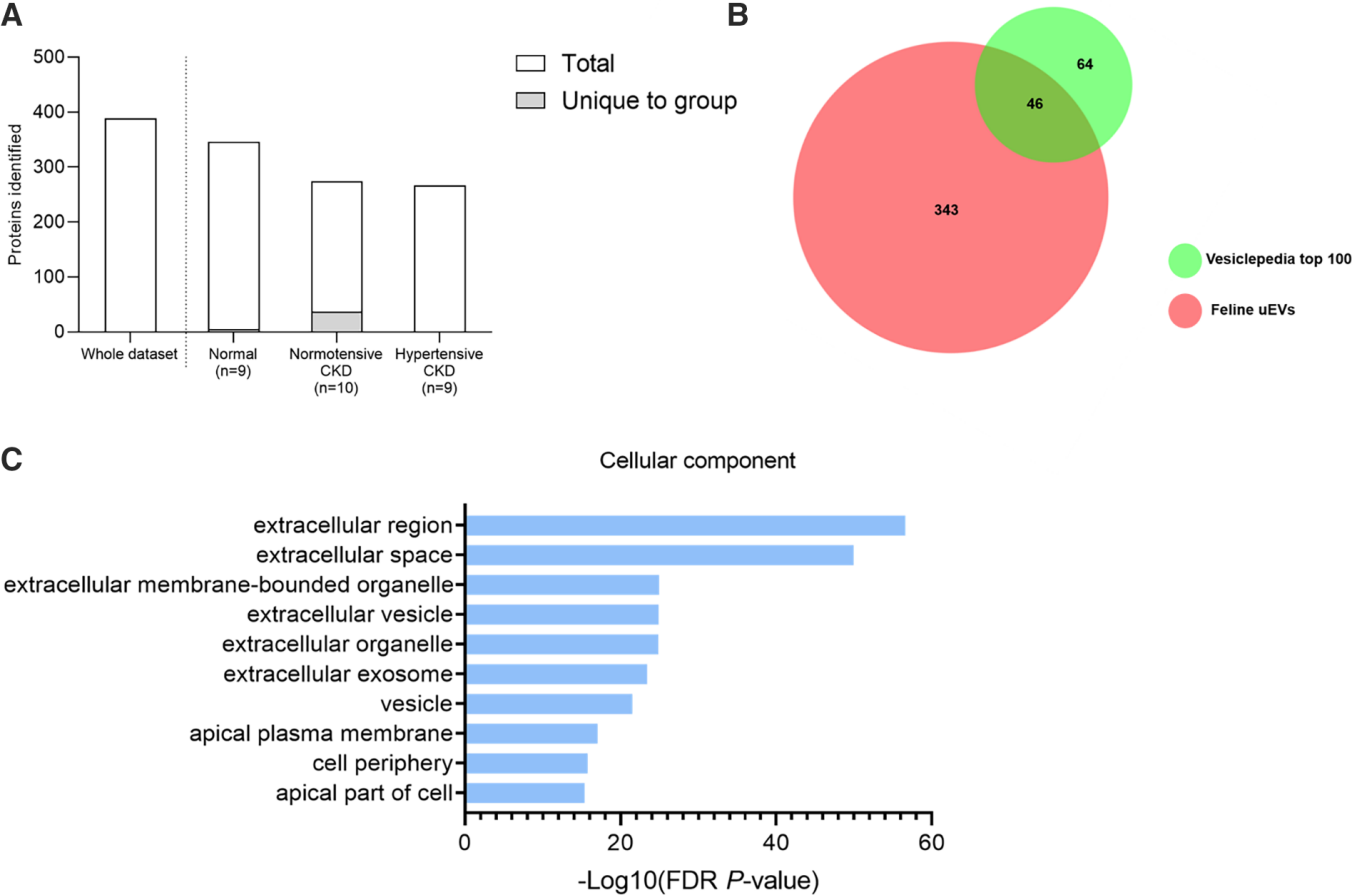
Bioinformatic characterisation of the uEV proteome obtained via LC‐MS/MS. (A) A total of 389 different proteins were detected across all samples, with 346 detected in uEVs from the normal category (n=9), 267 in uEVs from the normotensive CKD category (n=10) and 274 in uEVs from the hypertensive CKD (n=9) category. Of these proteins, five were unique to the normal category, 37 were unique to the normotensive CKD category and one was unique to the hypertensive CKD category. (B) Venn diagram of proteins identified in the feline uEV samples compared with proteins annotated in the Vesiclepedia Top100 EV proteins database (C) Gene ontology enrichment analysis by cellular component with −log10 (FDR P value) using the PANTHER classification system (v.14.0)

In total, 389 proteins were identified from all EVs isolated (Fig 5A). This set of uEV proteins was cross‐referenced with a publically available EV proteome database (“Vesiclepedia”) which demonstrated our current dataset contained 46 of the 100 most commonly reported EV proteins in other species (Fig 5B), including Alix (PDCD6IP), TSG101, tetraspanins (CD9, CD81), annexins, and heat shock proteins (Table 2; Kalra *et al*. 2012). In addition, proteins originating from all portions of the nephron were represented in the uEV proteome, including numerous apical membrane transporters (Table 3).

**Table 2:** Canonical extracellular vesicle marker proteins identified in the feline uEV proteome

Family	Identified protein
Annexins	ANXA2
	ANXA5
	ANXA7
	ANXA11
Tetraspanins	CD9
	CD81
Heat shock proteins	HSPA5
	HSPA8
Escort associated proteins	TSG101
	PDCD6IP
	SDCBP

uEV urinary extracellular vesicle

**Table 3:** Membrane proteins originating from the nephron identified in the feline uEV proteome

Nephron segment	Identified protein
Glomerulus	PODXL
	NPHS2
Proximal tubule	SGLT1
	SGLT2
	SLCO4C1
	SLC22A12
Loop of Henle	SLC12A1
Distal convoluted tubule	SLC12A3
Collecting duct	AQP2
	RHCG

uEV urinary extracellular vesicle

Gene ontology analysis revealed that proteins identified from feline uEVs were strongly enriched in the “extracellular membrane‐bounded organelle” (n=29, P=1.09E−25), “extracellular vesicle” (n=29, P=1.33E−25), “extracellular organelle” (n=27, P=1.36E−25), “extracellular exosome” (n=29, P=3.95E−24) and “vesicle” (n=82, P=2.94E−22) cellular components, further supporting effective isolation of the urinary EV fraction (Fig 5C). However, the proteome was also strongly enriched with proteins associated with the “extracellular region” (n=111, P=2.58E−57) and “extracellular space” (n=109, P=1.11E−50) suggesting the presence of non‐EV proteins within the uEV preparations.

**FIG 6:**
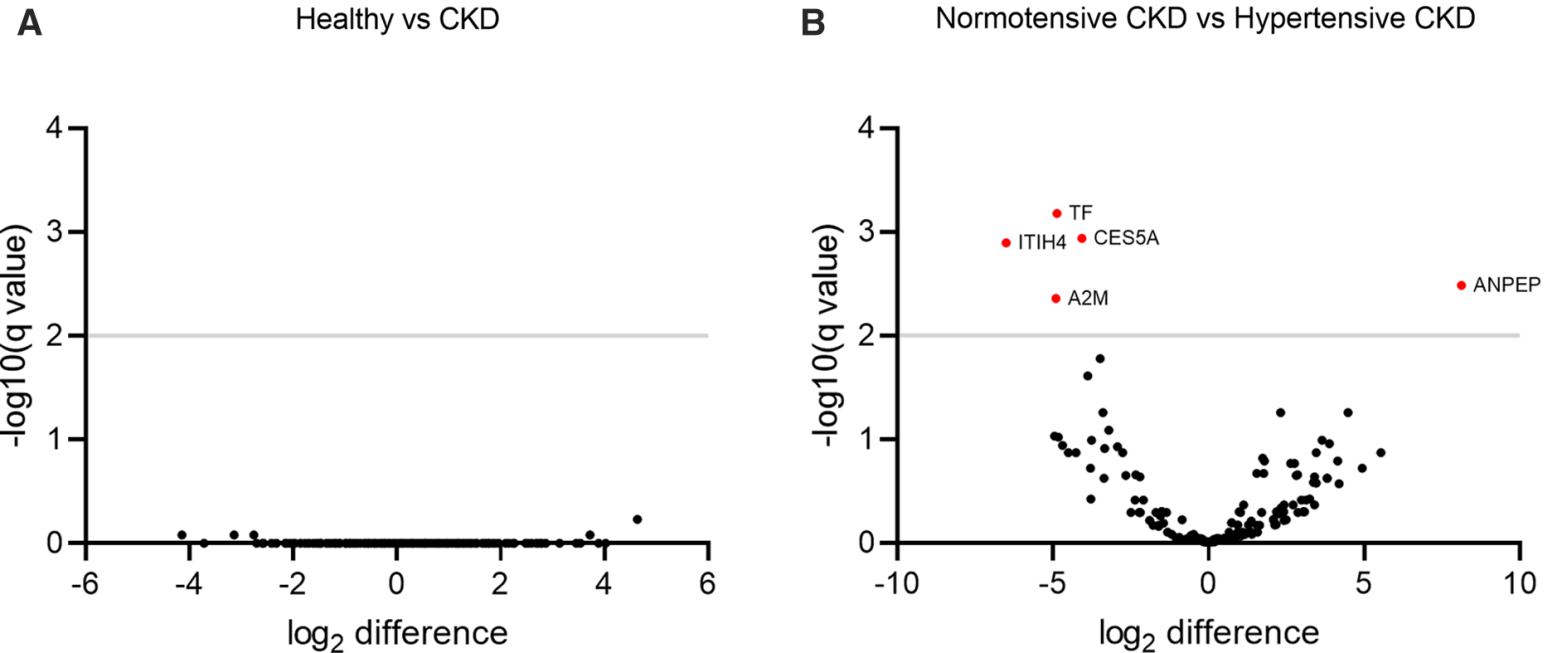
Volcano plot illustrating (A) differential protein expression in uEVs derived from healthy cats *versus* cats with diagnosed with CKD (normotensive and hypertensive CKD combined) and (B) differential protein expression in uEVs derived from cats from the normotensive CKD and hypertensive CKD category. Statistical significance was calculated using the unpaired Student's *t*‐test with Benjamini, Krieger and Yekutieli false discovery rate. The −log10 Q value (Benjamini, Krieger and Yekutieli corrected P value) is plotted against the log2 difference. Points above the non‐axial horizontal line represent proteins with significantly different abundances. There were no proteins significantly differentially expressed between the category of cats with normal renal function and the category of cats with CKD. ANPEP (aminopeptidase M, Q=0.0033) was significantly overexpressed in uEVs derived from the hypertensive CKD category when compared to the normotensive CKD category, whereas A2M (alpha‐2‐macroglobulin, Q=0.0044), CES5A (cauxin, Q=0.0012), ITIH4 (inter‐alpha‐trypsin inhibitor heavy chain 4, Q=0.0013) and TF (transferrin, Q=6.65×10^−4^) were significantly underexpressed

Outside of the most commonly detected proteins, there were significant number of missing proteins in the dataset (Fig S1), possibly because the concentrations of these proteins were below the limit of detection. There were no proteins differentially expressed when comparing the healthy category with the CKD category (Fig 6). When comparing the hypertensive CKD category with the normotensive CKD category, aminopeptidase M (ANPEP) was significantly overexpressed in uEVs derived from the hypertensive CKD category (Q=0.0033), whereas alpha‐2‐macroglobulin (A2M; Q=0.0044), cauxin (CES5A; Q=0.0012), inter‐alpha‐trypsin inhibitor heavy chain 4 (ITIH4; Q=0.0013) and transferrin (TF; Q=6.65×10^−4^) were significantly underexpressed (Fig 6). Differences and the standard error of the difference for these comparisons may be found in Data S1.

## DISCUSSION

The first goal of this study was to validate a methodology for the isolation of uEVs from feline urine. Whilst feline uEVs could be successfully isolated using all of the methods trialled, ultrafiltration followed by size exclusion chromatography resulted in the uEV preparations containing the highest number of particles and of the highest purity. Preparations obtained by ultrafiltration followed by size exclusion chromatography had the highest particle to protein ratio, which is recognised as a measure of EV purity (Webber & Clayton 2013), and contained visibly less background particulate matter (likely protein aggregates) on transmission electron microscopy. This result is in accordance with previous literature (Droste *et al*. 2021, Karttunen *et al*. 2021). EV preparations obtained via precipitation are recognised to be relatively impure, due to coprecipitation of contaminating proteins and lipoproteins (Konoshenko *et al*. 2018), and size exclusion chromatography has been demonstrated to result in the elution of purer EVs (Lobb *et al*. 2015). Large urine volumes were unable to be loaded onto the size exclusion chromatography columns, and therefore concentrating methods (ultrafiltration or precipitation) were required in order to reduce the volume before size exclusion chromatography in this study. Despite the superiority of the ultrafiltration followed by size exclusion chromatography method the preparations would still be classified in the “impure” range based upon particle: protein ratio (Webber & Clayton 2013). Therefore it is possible that the preparations contained a number of non‐EV associated proteins which could have acted as a confounding factor in the subsequent proteomics analysis. Further work to refine the purity of the feline uEV preparations would be beneficial.

This study also characterised the uEV population of cats with normal renal function, cats with normotensive CKD and cats with hypertensive CKD. The size distribution of the isolated EVs from cats in all categories was similar, and suggested that a large percentage were exosomes, a population of EVs derived from the endosomal system that are classically described as between 30 and 150 nm in size (Merchant *et al*. 2017). However, it is probable that the preparations also contained small microvesicles (100 to 1000 nm), which bud from the plasma membrane, and apoptotic bodies, shed from dying cells (Merchant *et al*. 2017). Previous studies in humans have yielded conflicting results regarding uEV numbers in patients with renal disease, with one study reporting no difference in number of urinary exosomes in patients with end stage renal disease *versus* controls (Dimuccio *et al*. 2014), whilst another found an increase in the number of small sized EVs in patients with diabetic nephropathy (Feng *et al*. 2021). There was no difference in uEV count per millilitre of urine or uEV number normalised to creatinine between categories in our study, suggesting that number of uEVs is not a useful biomarker of disease status in cats, and that uEV excretion rate is not substantially reduced in feline patients with reduced functional renal mass.

Analysis of the feline uEV proteome revealed numerous canonical EV markers, and there was significant overlap between the dataset and a publicly available database of commonly identified uEV proteins, supporting the identification of those particles identified on transmission electron microscopy and nanoparticle tracking analysis as EVs. Furthermore, the proteome was found to be strongly enriched in EV related cellular components by gene ontology analysis, although a significant number of proteins were associated with an extracellular derivation, possibly representing contamination by free protein. Previous studies have revealed that the urinary and reproductive tracts are the major source of urinary EVs in human patients, and the contribution of cells outside the urinary system is negligible (Svenningsen *et al*. 2020). In the feline EV preparations, membrane proteins from all nephron segments were detected via LC‐MS/MS, suggesting that, as in humans, a large proportion of EVs present in feline urine originate from the kidney. Proteins identified included podocyte markers alongside apical membrane transporter proteins from the proximal tubule, loop of Henle, distal tubule and collecting duct. The presence of these proteins raises the potential for uEVs to be utilised diagnostically as a “liquid biopsy”, considering that EVs derived from cells of the glomerulus and nephron associate with underlying kidney structural changes in humans (Turco *et al*. 2016), although further work is necessary to correlate the uEV population with renal pathology in cats.

In studies of humans with a variety of kidney diseases the EV proteome has been found to be altered, reflecting the underlying renal pathology (Turco *et al*. 2016). It was therefore unexpected that there were no proteins differentially expressed when comparing the healthy feline uEV proteome with the CKD uEV proteome in this study, which is also in contrast to studies of the wider urinary proteome in cats (Ferlizza *et al*. 2015). It is possible that true differences were masked by the relatively small category sizes in the current study. There were a significant number of missing values in the dataset, which was likely secondary to stochastic factors related to lack of LC‐MS/MS resolution in samples of low total protein content, in addition to genuine biological differences. It is also possible that proteolytic degradation could have occurred during the freeze‐thaw process that the urine samples underwent, and addition of protease inhibitor before freezing in future may increase yield. Therefore, the values available for comparison of individual proteins between categories were frequently smaller than the total category size. In addition, numerous factors are known to contribute to interindividual differences in uEV proteome in humans, including age and sex, and it is possible that similar confounding factors exist in feline patients (Turco *et al*. 2016, Erozenci *et al*. 2021).

When comparing the hypertensive CKD category to the normotensive CKD category, a number of differentially expressed proteins were detected. Aminopeptidase N (ANPEP) was found to be overexpressed in uEVs obtained from cats with hypertensive CKD. This enzyme is expressed in the brush border of tubular cells and plays a role in regulation of sodium transport as well as the metabolism of angiotensin III to angiotensin IV, and therefore may have a role in the regulation of blood pressure (Danziger 2008). To the author's knowledge, this is the first study to identify this enzyme as a potential mediator of feline hypertension, and additional studies are required in order to confirm and further elucidate its role in the pathogenesis of this condition. The biological relevance of the proteins significantly underexpressed in the hypertensive category is more challenging to determine. A2M is a protease inhibitor, CES5A is a carboxylesterase (and a major component of the feline urinary proteome), ITI4 is an acute phase protein involved in the inflammatory response and TF mediates the transport of iron. Considering that A2M, ITI4 and TF are all likely of blood rather than renal origin, and CES5A is secreted by proximal tubular cells, it is possible that these alterations are due to contamination of the uEV samples by extracellular proteins. However, a previous study investigating the uEV proteome of human patients with hypertension secondary to hyperaldosteronism identified altered expression of another acute phase protein, alpha‐1‐acid glycoprotein 1, and therefore these changes may have an as yet unknown biological relevance (Barros *et al*. 2021). Whether the alterations in protein expression documented in this study are involved in the pathogenesis of hypertension in CKD, or are an appropriate physiological response to hypertension, is unknown. Further studies are required to investigate the significance of these alterations, including whether similar changes are present in cats suffering from hypertension without concurrent CKD.

In summary, feline uEVs can be successfully isolated from the urine of cats with and without CKD, with ultrafiltration followed by size exclusion chromatography resulting in the purest uEV preparations. The uEV proteomes of cats with normal renal function, and cats with normotensive or hypertensive CKD could be comprehensively documented from 4 mL urine samples enabling confirmation of the presence of EV‐enriched proteins and canonical EV markers alongside markers representing every portion of the nephron were present. A number of differentially expressed proteins were discovered in cats with hypertensive CKD, which warrant further investigation into their utility as biomarkers or treatment targets.

### Conflict of interest

None of the authors has a financial or personal relationship with other people or organisations that could inappropriately influence or bias the content of the paper.

### Author contributions

**Jack Stephen Lawson:** Conceptualization (lead); data curation (equal); formal analysis (lead); funding acquisition (lead); investigation (lead); methodology (equal); project administration (lead); resources (equal); software (equal); validation (equal); visualization (equal); writing – original draft (lead); writing – review and editing (lead). **Harriet Syme:** Conceptualization (equal); formal analysis (supporting); funding acquisition (equal); investigation (equal); methodology (equal); resources (equal); supervision (equal); writing – review and editing (equal). **Robin Antrobus:** Methodology (equal); writing – original draft (supporting); writing – review and editing (supporting). **Jenni Karttunen:** Investigation (equal); methodology (equal); resources (equal); writing – review and editing (equal). **Sarah Stewart:** Methodology (equal); writing – review and editing (equal). **Fiona Karet Frankl:** Methodology (equal); resources (equal); writing – review and editing (equal). **Tim L Williams:** Conceptualization (equal); formal analysis (supporting); funding acquisition (equal); investigation (equal); methodology (lead); resources (equal); supervision (lead); validation (equal); writing – review and editing (equal).

## Supporting information


**Data S1:** Pilot experiment to determine an optimal methodology for the isolation of feline uEVs

**Figure S1:** Heat map illustrating coverage of the top 100 most abundant proteins in the feline uEV samples. Lighter colouration represents greater abundance, and darker colouration lower abundance. Outside of the most commonly detected proteins, there was significant number of missing values (represented by blank space), which lowered statistical power. Samples from cats in the healthy category are prefixed with an H, cats in the normotensive CKD category prefixed with NT‐CKD and cats in the hypertensive CKD category are prefixed HT‐CKD.
