# Supplementary material for: Urinary extracellular vesicles as a source of protein‐based biomarkers in feline chronic kidney disease and hypertension
Source: J Small Anim Pract. 2022 Jul 7;64(1):3–11. doi: 10.1111/jsap.13536 (PMC10084206; doi:10.1111/jsap.13536)
Supplement: Supplementary file 1 — Data S1: Pilot experiment to determine an optimal methodology for the isolation of feline uEVs [file JSAP-64-3-s002.docx]

|  | Mean Diff. | 95.00% CI of diff. | Adjusted P Value |
| --- | --- | --- | --- |
| Precipitation vs. Ultrafiltration+SEC | -546765905 | -940789289 to -152742520 | 0.0126 |
| Precipitation vs. Precipitation +SEC | -32289738 | -426313123 to 361733647 | 0.9659 |
| UF+SEC vs. Precipitation +SEC | 514476167 | 120452782 to 908499551 | 0.0166 |

**Supplementary data: Pilot experiment to determine an optimal methodology for the isolation of feline uEVs**

**Particle/protein ratio**

**Particles per mL**

|  | Mean Diff. | 95.00% CI of diff. | Adjusted P Value |
| --- | --- | --- | --- |
| Precipitation vs. Ultafiltration+SEC | -5270000000 | -6230309844 to -4309690156 | <0.0001 |
| Precipitation vs. Precipitation +SEC | 1377333333 | 417023490 to 2337643177 | 0.0108 |
| Ultrafiltration+SEC vs. Precipitation +SEC | 6647333333 | 5687023490 to 7607643177 | <0.0001 |

**Supplementary data: Characterisation of the uEV population in cats with normal renal function, normotensive CKD and hypertensive CKD**

**Particles per mL**

|  | Mean rank diff. | Significant? | Summary |
| --- | --- | --- | --- |
| Normal vs. Normotensive CKD | 3.544 | No | ns |
| Normal vs. Hypertensive CKD | 6.444 | No | ns |
| Normotensive CKD vs. Hypertensive CKD | 2.900 | No | ns |

**Particle/creatinine ratio**

|  | Mean rank diff. | Significant? | Summary |
| --- | --- | --- | --- |
| Normal vs. Normotensive CKD | 0.4222 | No | ns |
| Normal vs. Hypertensive CKD | 3.122 | No | ns |
| Normotensive CKD vs. HypertensiveCKD | 2.700 | No | ns |

**Proteomics analysis**

|  | P value | Mean of Hypertensive CKD (log2) | Mean of Normotensive CKD (log2) | Difference (log2) | SE of difference | t ratio | q value |
| --- | --- | --- | --- | --- | --- | --- | --- |
| TF | 0.000004 | 20.07 | 24.94 | -4.871 | 1.053 | 4.624 | 0.000665 |
| CES5A | 0.000015 | 23.89 | 27.96 | -4.071 | 0.9349 | 4.354 | 0.001150 |
| ITIH4 | 0.000025 | 17.91 | 24.42 | -6.504 | 1.534 | 4.240 | 0.001270 |
| ANPEP | 0.000084 | 23.82 | 15.69 | 8.128 | 2.058 | 3.949 | 0.003263 |
| A2M | 0.000142 | 16.76 | 21.66 | -4.904 | 1.283 | 3.821 | 0.004390 |
