# Supplementary figures and images for: Urinary extracellular vesicles as a source of protein‐based biomarkers in feline chronic kidney disease and hypertension
Source: J Small Anim Pract. 2022 Jul 7;64(1):3–11. doi: 10.1111/jsap.13536 (PMC10084206; doi:10.1111/jsap.13536)

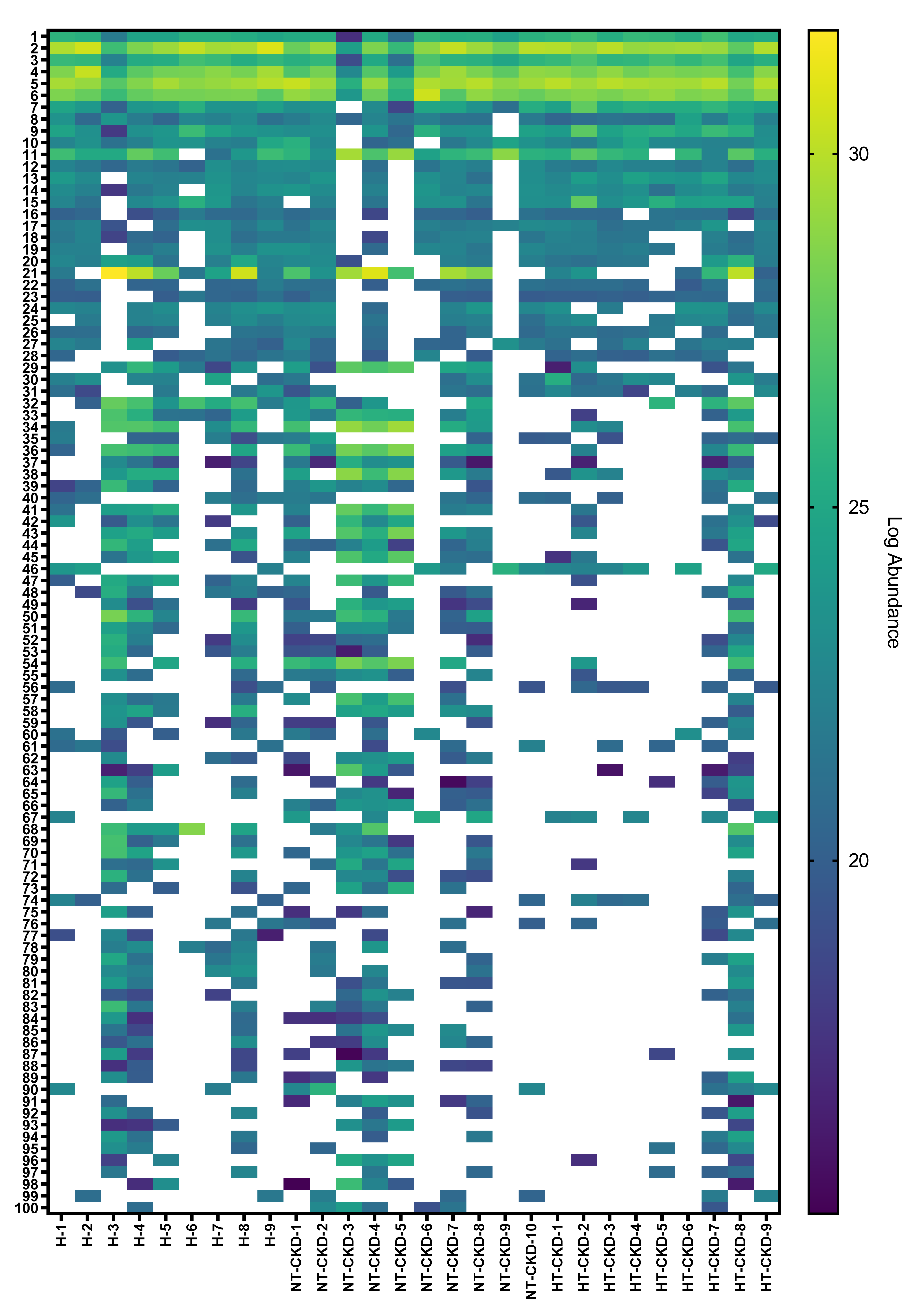

Supplement: Supplementary file 2 — Figure S1: Heat map illustrating coverage of the top 100 most abundant proteins in the feline uEV samples. Lighter colouration represents greater abundance, and darker colouration lower abundance. Outside of the most commonly detected proteins, there was significant number of missing values (represented by blank space), which lowered statistical power. Samples from cats in the healthy category are prefixed with an H, cats in the normotensive CKD category prefixed with NT‐CKD and cats in the hypertensive CKD category are prefixed HT‐CKD. [file JSAP-64-3-s001.tif]
